# Sponges

**Published:** 1893-09-02

**Authors:** 


### PRACTICAL DEPARTMENTS.

### SPONGES.

We have received a price list from Messrs. Cresswell Brothers, sponge importers, Red Lion Square, containing some information as to the various kinds of sponges, and the methods followed in their preparation for the market, which may interest our readers. Messrs. Cresswell, it appears, are the only English firm owning premises on the sponge fisheries, and from dealing thus directly with the public without the intervention of the middleman, they are enabled to supply sponges at a much lower rate than is usually the case.

A good deal of ignorance as to what sponges really are prevails among many people. We are told by Messrs. Cresswell that while some hold the conviction that the sponge belongs to the vegetable kingdom; other go further, and cherish the delusion that it is made by man," and that they have themselves been addressed as "Sponge Manufacturers," and that by a Government official. It may therefore be worth while to clear up these mistaken ideas by a few words anent the real nature of these wonderfully curious and beautiful structures, which are in such common everyday use amongst us. The sponge is the framework or skeleton of certain slime animals, low in the scale of nature, but nevertheless full of life. They are found at the bottom of the seas of the Grecian Archipelago, in the Mediterranean, along the Egyptian coast, and the Red Sea, while great numbers are found in the West Indies and on the coasts of Florida and Mexico, but by far the best are those of the Mediterranean. Their forms and colour are wonderfully varied. When torn from its home on the rocks or sea bottom, each sponge is seen to be covered and lined throughout with slime, made up of innumerable tiny cells, the whole constituting "one single sponge animal, which lives, breathes, feeds, grows, and gives forth young ones in its ocean home." This living slime is wrung from the flexible skeleton, and then follow various processes of bleaching, during which all organic impurities are removed. It will have been seen that some sponges are of a much lighter colour than others, and there is a general idea that the darker kind are "unbleached," and therefore more durable. But this is a mistake. All sponges are bleached, but those of a lighter colour have been subjected to a special chemical process, which has a particularly purifying effect.

There are many varieties of sponges; the two best-known kinds being the Turkey and the honeycomb. But it is not perhaps generally known that Cuban sponges are considerably less expensive than the two first mentioned, and though somewhat coarser, are in most respects quite as good. The difference in price arises from the fact that Cuban and West Indian sponges are more easily obtained than those from the Mediterranean, owing to their growing in shallower water. They are chiefly reached through the means of diving apparatus, but native divers are sometimes employed, and sponges can also be fished by the harpoon, but the last method is likely to result in damage to the sponge. Messrs. Cresswell's principal sponge-fishing station is at Ægina (Greece), where they have huge warehouses, and where the processes of sorting, trimming, &c., are carried on. Nurse readers will be interested to know that No. 1, Red Lion Square, one of the London warehouses of this firm, was originally a hospital, instituted by Miss Nightingale, just after the Crimean War, for "diseases of the legs, ulcers, &c." Here are now stocked sponges of all shapes and kinds, and we would suggest that would-be purchasers should pay a visit to these headquarters, and judge for themselves of the comparative merits of each.

